# The use of LiDCO based fluid management in patients undergoing hip fracture surgery under spinal anaesthesia: Neck of femur optimisation therapy - targeted stroke volume (NOTTS): study protocol for a randomized controlled trial

**DOI:** 10.1186/1745-6215-12-213

**Published:** 2011-09-28

**Authors:** Matthew D Wiles, William JD Whiteley, Chris G Moran, Iain K Moppett

**Affiliations:** 1 Division of Anaesthesia and Intensive Care, University of Nottingham, University Park, Nottingham, NG7 2RD, UK; 2 Department of Anaesthesia, Nottingham University Hospitals NHS Trust, Nottingham, NG7 2UH, UK; 3 Department of Trauma and Orthopaedics, Nottingham University Hospitals NHS Trust, Nottingham. NG7 2UH, UK; 4 Department of Anaesthesia, Sheffield Teaching Hospitals NHS Foundation Trust, Sheffield. S10 2JF, UK

## Abstract

**Background:**

Approximately 70,000 patients/year undergo surgery for repair of a fractured hip in the United Kingdom. This is associated with 30-day mortality of 9% and survivors have a considerable length of acute hospital stay postoperatively (median 26 days). Use of oesophageal Doppler monitoring to guide intra-operative fluid administration in hip fracture repair has previously been associated with a reduction in hospital stay of 4-5 days. Most hip fracture surgery is now performed under spinal anaesthesia. Oesophageal Doppler monitoring may be unreliable in the presence of spinal anaesthesia and most patients would not tolerate the probes. An alternative method of guiding fluid administration (minimally-invasive arterial pulse contour analysis) has been shown to reduce length of stay in high-risk surgical patients but has never been studied in hip fracture surgery.

**Methods:**

Single-centre randomised controlled parallel group trial. Randomisation by website using computer generated concealed tables. Setting: University hospital in UK. Participants: 128 patients with acute primary hip fracture listed for operative repair under spinal anaesthesia and aged > 65 years. Intervention: Stroke volume guided intra-operative fluid management. Continuous measurement of SV recorded by a calibrated cardiac output monitor (LiDCOplus). Maintenance fluid and 250 ml colloid boluses given to achieve sustained 10% increases in stroke volume. Control group: fluid administration at the responsible (blinded) anaesthetist's discretion. The intervention terminates at the end of the surgical procedure and post-operative fluid management is at the responsible anaesthetist's discretion. Primary outcome: length of acute hospital stay is determined by a blinded team of clinicians. Secondary outcomes include number of complications and total cost of care.

Funding NIHR/RfPB: PB-PG-0407-13073.

**Trial registration number:**

Trial registration: Current Controlled Trials ISRCTN88284896.

## Background

Approximately 70,000 patients per year undergo surgery for repair of a fractured hip in the United Kingdom (UK) [1], at a cost of £25,424 per patient [2]. Hip fracture is associated with a high mortality (approximately 9% at 30 days postoperatively)[3] and a considerable length of acute hospital stay (median stay 26 days) [4]. Mortality and length of stay in hospital have remained relatively constant over recent years. The majority of patients with hip fracture are elderly women (median age 81 years, 73% female) [2], and the number of these patients will likely increase in the future in line with the changes in the United Kingdom population [5-7].

Patients with hip fracture suffer from a variety of fluid losses following their injury. They spend a variable amount of time unable to drink or eat, due to immobility at the time of injury and fasting prior to surgery. The fracture itself will haemorrhage internally to a variable degree and there may clinically important blood loss both during and after surgery [8]. Many elderly patients are taking medications which promote fluid loss (mainly diuretics) [9]. For these reasons most patients are given intravenous fluid on admission. During surgery the anaesthetist usually gives intravenous fluids based upon clinical judgement of all the above factors and clinical signs such as heart rate and blood pressure.

The amount of intravenous fluid patients are given before, during and after surgery, may have prolonged effects on outcome. Studies in non-orthopaedic surgery suggest that protocol driven (or goal directed) intra-operative fluid administration is associated with improved outcome and reduction in hospital stay [10,11]. The aim of goal-directed fluid therapy is to optimise the amount of blood in the veins filling the heart and therefore optimise stroke volume (volume of blood leaving the heart with every heart beat), cardiac output (volume of blood pumped by the heart per minute) and thereby oxygen delivery to the tissues. A number of studies suggest that derangement of cardiac output is one factor that is strongly associated with post-operative complications and death [12]. Previous studies of patients undergoing surgery for fractured hip have shown that using a Doppler ultrasound probe placed in an anaesthetised patient's oesophagus to measure the velocity of blood flow in the aorta to help guide fluid administration, resulted in a reduced hospital stay from 17 to 12 days [13-15]. Administering a fixed dose of additional fluid without cardiac output monitoring does not seem to be of benefit [16]. However, many patients receive a spinal anaesthetic for hip surgery, a technique which is associated with a lower risk of heart attack, confusion and low blood oxygen levels compared to general anaesthesia [17]. During spinal anaesthesia, the patient remains awake or lightly sedated and therefore would be unlikely to tolerate an ultrasound probe in their oesophagus [18]. The ultrasound measurements of cardiac output may also be unreliable when a spinal anaesthetic is used [19].

An alternative to the Doppler ultrasound probe is estimation of cardiac output by analysis of the waveform of the arterial blood pressure pulse. This technique has become more practical and widespread in recent years due to advances in technology. It is a minimally invasive technique, well tolerated in awake or sedated patients, which analyses arterial pressure changes during the cardiac cycle, allowing calculation of stroke volume and cardiac output [20]. This technique has been used to guide the volume of intravenous fluid administered to patients at high risk of complications after major surgery, and when used resulted in a shorter hospital stay [21]. It has never been studied in patients having hip fractures repaired to the author's knowledge, based on literature review (including an updated systematic search based upon Parker [16], and discussion with peers). In the period since the original papers demonstrating benefits of goal-directed fluid therapy in hip fracture, other practices have also changed. More patients are given intravenous fluids as soon as they are admitted, the time to surgery has decreased and more patients have surgery performed under spinal anaesthesia. It is therefore appropriate to investigate whether goal-directed fluid management is of benefit in contemporary hip fracture care.

In addition, to the authors' knowledge the changes in the heart and blood vessels produced by a spinal anaesthetic in patients with fractured hips have never been characterised fully. Patients with a fractured hip are often elderly with numerous associated medical problems. Knowledge of how spinal anaesthetics affect the heart and blood vessels in this high risk population may help guide how to best maintain the patient's blood pressure and blood flow during surgery.

## Methods

### Study Objectives

#### Primary Aims

To investigate whether stroke volume (SV) guided fluid therapy for patients undergoing fractured hip surgery under a spinal anaesthetic affects:

1) the time the patient spends in acute care in hospital;

#### Secondary Aims

2) To investigate if using SV guided fluid therapy a patient receives while having their fractured hip repaired under a spinal anaesthetic affects:

a. the time until the patient is medically well enough to be discharged from hospital;

b. the number of postoperative complications;

c. the total amount of intravenous fluid given during surgery;

d. the total cost of care.

3) To characterise the changes in the way the heart and blood vessels work after a spinal anaesthetic, as measured by minimally-invasive cardiac output monitoring.

### Study Design

This is a prospective, single-centre, randomised, parallel group controlled trial conducted at the Queens Medical Centre campus of Nottingham University Hospitals, Nottingham, UK. Study recruitment commenced on 9^th ^September 2009 when the first patient was randomised. Recruitment is ongoing (August 2011).

### Randomization and Blinding

Randomisation (on a one-to-one allocation basis) is via a password-protected web based randomisation service provided by the Clinical Trials Support Unit and the sequence not revealed until datalock. To achieve similar numbers and balance on risk, patients will be stratified according to predicted 30-day mortality (2 levels). The Nottingham Hip Fracture Score [22-24], is used to identify patients with low (≤ 10%) or high (> 10%) risk of mortality within 30-days. Subjects are randomised on arrival in the anaesthetic room to minimise the risk of medical or surgical cancellation after randomisation.

Patients, orthopaedic surgeons and ward staff are blinded to the treatment intervention. The operative attending anaesthetist is not blinded to the treatment arm, but is blinded to the cardiac output measurements for the control arm. Postoperative data collection is by a research nurse blinded to treatment allocation. The decision that a patient is medically fit for discharge is made by the multiprofessional team when all are satisfied that the participant has no ongoing needs for acute hospital care. This team is blinded to participant allocation.

### Selection and withdrawal of participants

#### Recruitment

Participants are identified by their presence on surgical lists and are recruited from the trauma wards. The investigator informs the participant or their nominated representative (other individual or other body with appropriate jurisdiction), of all aspects pertaining to participation in the study. Participation in the study is for the duration of the anaesthetic and surgical procedure, which is approximately two hours. The study intervention is complete at the end of the surgical procedure, though all participants have a follow-up visit the next day to ensure no problems have arisen. The medical notes are reviewed following hospital discharge for in-hospital complications and medication use.

Participants are informed that entry into the trial is voluntary and that they are free to withdraw at anytime without effect on subsequent care. Data on time to discharge and postoperative mortality are collected routinely and separate from this study. These outcome data are therefore available for all randomised participants.

Included in the trial are adults incapable of providing their own consent, since a large proportion of potential subjects will be acutely or chronically confused (around 30%). Exclusion of these patients from the trial would severely limit the applicability of results to clinical practice, since these patients are more likely to die, remain in hospital for longer, and have more complications [22,25-27].

Patients from whom consent cannot be obtained due to a language barrier are not recruited to the study. This is due to the emergency nature of the surgery meaning that it would not be practical to arrange for the provision of translation services. In practice this is a very small proportion of the total elderly population in the study centre.

### Inclusion criteria

1. Patients listed for surgical repair of fracture neck of femur under spinal anaesthesia

2. Aged > 65 years

### Exclusion criteria

1. Planned general anaesthetic

2. Severe valvular heart disease previously shown on echocardiography (reduces reliability of monitor measurements)

3. Taking lithium (interferes with calibration of monitor)

4. Multiple injuries requiring operative management

5. Revision surgery or total hip arthroplasty for fractured neck of femur (a different patient/surgical population)

6. Not admitted to hospital through the emergency department

### Informed consent

The capacity for consent is assessed routinely by the orthopaedic team, who decide whether the patient is competent to provide consent for the surgical procedure. If they deem the patient is unable to consent for their surgery, then the patient will be deemed incapable of providing consent to enter the study, and assent from their next of kin is sought (or nominated consultee). The capacity for consent may fluctuate in the population being studied. A member of the research team also performs an additional check of the participant's ability to consent, immediately prior to starting the study. All members of the research team are trained at taking consent in accordance with ICH-GCP guidance [28].

All patients are visited postoperatively. If the patient had had a transient impairment of their ability to consent, which has subsequently resolved, retrospective consent is sought from the patient to use their data in the study. If the patient declines, date of discharge and postoperative mortality data are collected as part of routine clinical management and so will be available for analysis. The same would occur if contact with the next of kin pre-operatively had not been possible. If the next of kin subsequently declined assent, the patient data would once again be withdrawn from the study.

### Measurement of cardiac output

Monitoring of the key cardiovascular variables of blood pressure and cardiac output (volume of blood pumped by the heart per minute) is common in patients at high risk of intra-operative and postoperative complications. One such method employed is the LiDCOplus (LiDCO Ltd, Cambridge, UK). This device allows calibrated, continuous, real-time monitoring of cardiac output on a beat-to-beat basis. The LiDCOplus cardiac output method of measurement is based upon the bolus indicator dilution method of measuring cardiac output, which is the standard method of measuring cardiac output [29,30]. The only equipment required is a peripheral cannula in a vein and an arterial line (a small cannula inserted into an artery that is routinely used in high risk patients).

A small dose of lithium chloride (0.15 mmol) is injected through the venous cannula. This is a very small dose of lithium, which has no known pharmacological effect [31]. Lithium has been used for the measurement of cardiac output for many years without any side effects being reported. A lithium concentration-time curve is measured by withdrawing 5 ml of blood past a lithium sensor attached to the patient's existing arterial line and the monitor then calculates the cardiac output from the area of the primary dilution curve [32,33].

After this initial calibration, beat-to-beat measurements of cardiac output are made by analysing changes in the arterial pressure waveform. The device does not need recalibrating for at least 8 hours [29]. The LiDCOplus monitor provides values for cardiac output, systemic vascular resistance, mean arterial pressure, heart rate and left ventricle stroke volume. These are adjusted for patient weight and height. As measurements of these variables are made on a beat-to-beat basis, the LiDCOplus is an ideal device for measuring changes in cardiovascular variables to interventions such as fluid boluses.

This method of cardiac output measurement has been shown to be as accurate as the thermodilution method employed by pulmonary artery flotation (Swan-Ganz) catheters [32,33] and avoids the complications associated with their insertion and use. The LiDCOplus system is also quicker and easier to set up then than pulmonary artery catheter systems, which have previously been thought of as the gold standard of cardiac output measurement devices. The LiDCOplus monitor remains accurate even if the arterial waveform becomes over- or under-damped [34] or if regional anaesthetic techniques (such as spinal or epidural anaesthesia) are used [35].

### Study Intervention

On arrival in the anaesthetic room, all participants have an arterial line sited at the wrist using local anaesthetic and an arterial blood sample taken (to determine sodium and haemoglobin concentrations for calibration of the LiDCOplus monitor). A cannula is inserted into a peripheral vein for calibration purposes and to allow fluid administration, if there is not an adequate one already in place. The arterial line is then connected to the LiDCOplus machine and calibration performed according to the manufacturer's guidelines, using a bolus intravenous injection of lithium [36]. Routine anaesthetic monitoring is instituted by the attending anaesthetist. The spinal anaesthetic is then performed and the patient remains in the anaesthetic room for at least 10 minutes to allow collection the post-spinal anaesthetic data from the LiDCOplus monitor. The patient is then taken into theatre once the anaesthetist is satisfied that the spinal anaesthetic is adequate for surgery.

The management of fluids during the operation differs between the study group and the control group. The control group have the type, amount and rate of their intravenous fluids determined by the anaesthetist, in accordance with their usual practice. The anaesthetist is blinded to the LiDCOplus measurements by placing the LiDCO screen where it is not visible and turning off the arterial waveform display. All intra-operative fluid boluses and drugs administered by the anaesthetist, including timings, are recorded by the Investigator.

**Figure 1:**
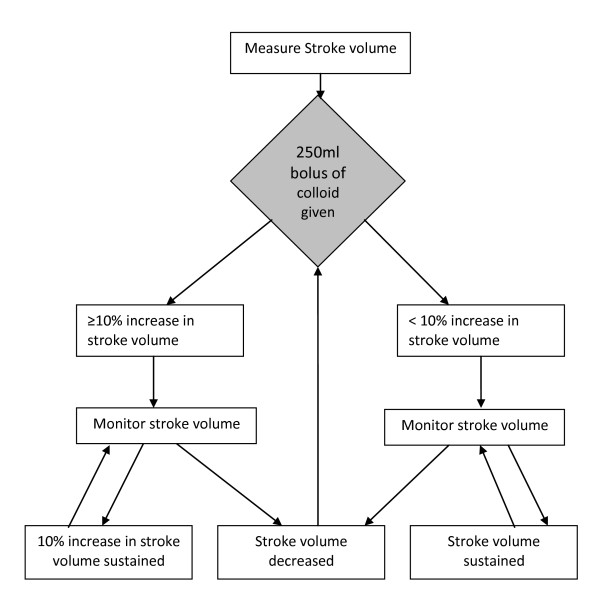
**Flow chart for administration of colloid boluses in the intervention group**. Changes in stroke volume are used to guide the administration of colloid boluses. The aim is give colloid boluses to achieve cardiac filling near the top of the Starling curve. The algorithm is interpreted in the light of clinical conditions.

The study group have their fluids given according to the study protocol, based upon LiDCOplus measurements. The participants in this group receive a maintenance fluid of compound sodium lactate solution at a rate of 1.5 ml kg^-1 ^h ^-1^. In addition they may be given 250 ml boluses of a colloid fluid, determined by the LiDCOplus measurements of stroke volume. Fluid boluses are given until the stroke volume to achieve a sustained 10% increase from the baseline (pre-operative) readings. Further boluses are given subsequently should the stroke volume fall by clinically important amounts at any time during the operation (see Figure 1). If the attending anaesthetist feels there are clinical reasons to give more or less fluid they can do so. If blood transfusion is required packed red cells are given as the colloid. Otherwise the colloid given is at the discretion of the anaesthetist. Discretionary actions of the attending anaesthetist are recorded.

The intervention is complete at the end of surgery. At this point the LiDCOplus monitoring is stopped, and the arterial cannula removed. The total amount of fluid is recorded for both groups. Postoperative fluids prescribed for participants are at the discretion of the anaesthetist.

### Standard care

All patients are admitted to dedicated trauma wards and cared for in accordance with UK 'Best Practice Tariff' [37]. This includes: assessment by orthogeriatricians; operation within 36 hours of admission; bone health assessment. All patients are cared for under a hip fracture care pathway which involves rapid assessment and admission from the emergency department; intravenous crystalloid infusions from time of admission and multiprofessional care and discharge planning. Operations are performed in dedicated trauma theatres by consultants in anaesthesia and orthopaedic trauma or senior trainees. The Queen's Medical Centre has undertaken continuous, systematic audit of its hip fracture care since 2001 [22-24].

### Cost Analysis

Data on cost of treatment will be calculated from drug use, investigations (diagnostic imaging, ECGs, blood tests) and from the clinical record, which includes standardised costs for physiotherapy, occupational therapy and discharge planning. The cost of additional monitoring required for each patient will also be taken into consideration in the cost analysis. Data will be presented in terms of total non-operative costs, costs per day and excess costs attributable to treatment group.

### Statistics

Data will be analysed by the research team in conjunction with a medical statistician, using latest versions of StatXact software. There will be no interim analysis.

From previous examination of the Nottingham Hip Fracture Database (which has over 7000 patients) it is expected that the primary outcome, length of stay (LOS), will be normally distributed following a log transformation. Following transformation these data will be analysed using ANOVA using the stratification risk categories. The primary outcome measure is time until declared medically fit for discharge. Length of acute hospital stay, superspell (total time in hospital including rehabilitation) will be analysed similarly.

The secondary outcomes of complications, residence and place of discharge will be treated as categorical and differences between the groups will be examined using loglinear analysis. Mortality outcome will be analysed using Kaplan-Meier mortality curves and log rank tests..

All secondary variables will the presented using descriptive statistics. In addition to the calculated values, confidence intervals and odds-ratios will be presented when appropriate. All clinical information including all adverse events will be presented in full. All secondary analyses will be interpreted with caution as the sample size calculation is based on the primary outcomes only. However, the level of power associated with secondary results will be investigated.

### Sample size and justification

Previous studies of optimisation strategies have demonstrated approximately 30% reduction on acute length of stay or time until medically fit to discharge and approaches towards 50% reductions in complications in survivors.

Length of acute hospital stay in our unit is consistently around 17 days (SD 4.9 days), so to find a 3 day reduction in LOS in survivors would require 58 patients in each group with 90% power and α = 0.05. Assuming a 10% drop out rate (conversion to general anaesthesia, operative cancellation after randomisation etc.) we aim to randomize 64 subjects in each group. This would also provide the same power to find a 50% reduction in complication rates between the two groups. The study will not be powered to demonstrate differences in mortality (a 50% reduction in 30-day mortality from 10% to 5%, would require around 450 patients in each group).

### Definition of datasets analysed

Safety set: All randomised participants who received LiDCOplus monitoring during surgery receive at least one dose of the study drug.

Full Analysis set: All randomised participants, who received LiDCOplus monitoring during surgery and for whom at least death and discharge date are available.

Per protocol set: All participants in the Full Analysis set who are deemed to have no major protocol violations that could interfere with the objectives of the study.

Efficacy will be assessed on both the full analysis set and the per protocol set; not the Intention-to-treat set.

Safety summaries will be performed on the safety set

### Reporting of adverse events

All adverse events will be recorded and closely monitored until resolution, stabilisation, or until it has been shown that the study treatment is not the cause. Participants will be asked to contact the study site immediately in the event of any serious adverse event. The Chief Investigator shall be informed immediately of any serious adverse events and shall determine seriousness and causality in conjunction with any treating medical practitioners.

All treatment related serious adverse events will be recorded and reported to the Research Ethics Committee as part of the annual reports. Unexpected serious adverse events will be reported to the Research Ethics Committee and Sponsor within the relevant timeframes. The Chief Investigator shall be responsible for all adverse event reporting.

### Ethics Committee and Regulatory Approval

The trial will be conducted in accordance with the ethical principles that have their origin in the Declaration of Helsinki, 1996 [38]; the principles of Good Clinical Practice [28], and the Department of Health Research Governance Framework for Health and Social care, 2005 [39].

Approval was obtained from the Nottingham Research Ethics Committee (Reference Number 08/H0403/129) and from the National Health Service Research and Development department (Reference Number 08AN004). The study was also registered with the National Institute for Health Research (Reference Number PB-PG-0407-13073) and IRCTN: 88284896 (29 April 2010). The study is ongoing with the first participant randomised on 9 September 2009.

## Discussion

Studies into peri-operative management of patients undergoing surgery for hip fracture are lacking [40]. Anaesthetic interventions in the peri-operative management of hip-fracture tend to occur within a short period of time owing to the emergency nature of the surgery, with surgical fixation recommended within 48 hours of hospital admission [41]. This results in important time constraints in the practical aspects of study conduct and participant recruitment to any potential trial. Gaining informed consent is consequently problematic, an issue compounded by the large proportion of acutely and chronically confused patients who present with hip fracture: exclusion of such patients from the trial would severely limit the applicability of the results to clinical practice and thus measures are taken to allow their participation, including the involvement of the next of kin or responsible consultant.

There is no single outcome measure that is accepted as the 'gold-standard' in hip fracture research. Length of stay has financial implications and may act as a surrogate for medical complications [9] but is strongly affected by local practices and availability of rehabilitation and social services. Mortality is a definite end-point. However some authors suggest that 50-75% of hip fracture deaths following surgery are inevitable [42], so mortality may not be amenable to significant reduction. Various studies have used mobility measures [43,44] but by and large these are surrogates for actual post-discharge function, and there is little consistency between studies. Discharge destination again has large financial and social implications but is strongly affected by local practices, and is irrelevant to the sizeable minority of patients who are admitted from nursing home care [24].

Most studies into peri-operative interventions for fractured hip repair report outcomes focusing on length of hospital stay or 30-day postoperative mortality. In addition to length of hospital stay, our study includes time to being medically fit for discharge as a primary outcome measure. Both these outcome measures are collected by members of the research team blinded to treatment allocation. Although both primary outcomes are relevant, they are affected by many other clinical and non-clinical factors, including availability of physiotherapy services, pre-fracture morbidity and mobility, and family/social services support. The secondary end-point in our study of post-operative complications is aimed more specifically at determining the effects of the anaesthetic intervention under investigation in the study.

Although the original studies of stroke volume guided fluid therapy showed clinically important results there has been variable uptake of the technology in UK clinical practice. This may reflect changes in clinical practice, such as earlier surgery and prescription of routine intravenous fluids before surgery, which might be expected to reduce the fluid deficit by the time the patients arrive in theatre. In addition the technology used in the original studies is not suitable for use in patients who are awake. We therefore felt that it was timely to investigate this approach in contemporary clinical practice. The choice of cardiac output monitoring (LiDCOplus) in our study was pragmatic based upon validity and availability. At this time there are a limited number of calibrated minimally invasive devices suitable for this purpose. Non- or minimally invasive devices that do not require calibration are available and may be used for goal-directed intra-operative fluid management, provided the alternative system has the ability to accurately predict fluid responsiveness.

The potential implications of our results are important. Approximately 80,000 patients per year undergo hip fracture surgery in the UK. Ward costs contribute 84% of the total cost of hospital expenditure of patients undergoing such a procedure, at a rate £430 per patient per day [45]. If stroke volume guided fluid management results in a reduction in hospital stay of 5 days, as demonstrated using the Doppler ultrasound probe [13,14], the saving could be around £2,150 per patient. This equates to a potential national saving in the UK of around £150 million per year. The reduction in duration of hospital stay is therefore one potential way of minimising expenditure following hip fracture.

Hip fracture is likely to become an increasing health issue within the UK [6,7], with associated economic implications, and a high mortality and morbidity allied to patients who suffer such injury. Despite this there is a limited evidence base with regards to guidance of best practice. Our study aims to augment this evidence base and improve the peri-operative management of patients presenting with fractured hip.

## Competing interests

This study is funded by the National Institute for Health Research. The authors do not receive any reimbursement or financial benefits and declare that they have no competing interests.

## Authors' contributions

MDW is the protocol author and is an investigator. WJDW is an investigator in the study and drafted the manuscript. CGM is an investigator and has revised the manuscript; he set up and supervises the trauma audit team. IKM is the chief investigator, grant holder and revised the manuscript.
